# Ketosis, ketogenic diet and food intake control: a complex relationship

**DOI:** 10.3389/fpsyg.2015.00027

**Published:** 2015-02-02

**Authors:** Antonio Paoli, Gerardo Bosco, Enrico M. Camporesi, Devanand Mangar

**Affiliations:** ^1^Nutrition and Exercise Physiology Laboratory, Department of Biomedical Sciences, University of PadovaPadova, Italy; ^2^Department of Surgery, University of South FloridaTampa, FL, USA; ^3^TEAMHealthTampa, FL, USA; ^4^Tampa General HospitalTampa, FL, USA

**Keywords:** ketones, ketogenic diet, hunger, brain, hypothalamus, appetite

## Abstract

Though the hunger-reduction phenomenon reported during ketogenic diets is well-known, the underlying molecular and cellular mechanisms remain uncertain. Ketosis has been demonstrated to exert an anorexigenic effect via cholecystokinin (CCK) release while reducing orexigenic signals e.g., via ghrelin. However, ketone bodies (KB) seem to be able to increase food intake through AMP-activated protein kinase (AMPK) phosphorylation, gamma-aminobutyric acid (GABA) and the release and production of adiponectin. The aim of this review is to provide a summary of our current knowledge of the effects of ketogenic diet (KD) on food control in an effort to unify the apparently contradictory data into a coherent picture.

## Introduction

Hunger and satiety are two important mechanisms involved in body weight regulation. Even though humans can regulate food intake by will, there are systems within the central nervous system (CNS) that regulate food intake and energy expenditure. This complex network, whose control center is spread over different brain areas, receives information from adipose tissue, the gastrointestinal tract (GIT), and from blood and peripheral sensory receptors. The actions of the brain's hunger/satiety centers are influenced by nutrients, hormones and other signaling molecules. Ketone bodies are the major source of energy in the periods of fasting and/or carbohydrate shortage and might play a role in food intake control.

## Hypothalamic control of feeding/appetite/hunger

### Role of nutrients in food intake control

The hypothalamus is the brain's main center responsible for hunger/satiety (H/S) control. In the theory that Mayer proposed more than 60 years ago, he assigned a central role to glucose levels in the H/S control: the so-called “glucostatic theory” (Mayer, 1955). Mayer suggested that depletion of carbohydrate availability leads to hunger, and the hypothalamic centers with receptors sensitive to glucose levels might be involved in the short-term regulation of energy intake (Mayer, 1955). The “feeding center” in the lateral hypothalamic area (LHA), according to the glucostatic theory, reacts to the between-meal fall of blood glucose and stimulates food intake. The LHA contains glucose-inhibited neurons that are stimulated by hypoglycemia, a process crucial to mediating the hyperphagia normally induced by hypoglycemia. The subsequent post-prandial hyperglycemia activates the “satiety center” in the ventromedial hypothalamus (VMH), which contains glucose-excited neurons and inhibits both “feeding center” and food intake.

In 1953, Kennedy proposed the lipostatic hypothesis suggesting that lipid metabolites could also be involved in food regulation (Kennedy, 1953), and in 1956, Mellinkoff studied the effects of protein metabolism suggesting an aminostatic hypothesis (Mellinkoff et al., 1956).

Glucose-sensitive neurons have been identified in a number of CNS regions including the metabolic control centers of the hypothalamus. Medeiros et. al. have used patch-clamp electrophysiology to examine whether neurons in a specific specialized region known as the subfornical organ (SFO), an area where the blood-brain barrier is not present, are also glucose sensitive or not. These experiments demonstrated that SFO neurons are glucose-responsive and that SFO is an important sensor and integrative center of circulating signals of energy status (Medeiros et al., 2012).

But comprehensive transcriptional profiling of glucose-sensing neurons is challenging, as glucokinase (Gck) and other key proteins that transduce glucose signals are expressed at low levels. Glucose also exerts a hormonal-like action on neurons; electrophysiological recordings demonstrated, for example, that hypoglycemia activates growth hormone-releasing hormone (GHRH) neurons, suggesting a mechanistic link between low blood glucose levels and growth hormone release (Stanley et al., 2013).

Nutrient-sensitive neurons reacting to glucose but also to fatty acids (FAs) concentrations are present at many sites throughout the brain and may play a key role in the neural control of energy and glucose homoeostasis. Central administration of oleate, for example, inhibits food intake and glucose production in rats. This suggests that daily variations in plasma FA concentrations could be detected by the CNS as a signal that contributes to the regulation of energy balance (Moulle et al., 2014).

Even though intracellular metabolism and activation of the ATP-sensitive K^+^ channels appear to be necessary for some signaling effects of FAs, a great amount of the FA responses in the ventromedial hypothalamic neurons are mediated by interactions with fatty acid translocase (FAT)/CD36. Translocase is a FA transporter/receptor that activates downstream signaling even in the absence of intracellular metabolism (Moulle et al., 2014).

The classical unified model is based on the role of the three metabolic substrates: lipids, glucose and protein/amino acids in maintaining the nutritional status throughout the corresponding loci in the CNS, but there are many other signals and brain targets (Williams et al., 2001).

### Role of the neuroendocrine system in food intake control

More recently, other hypothalamic appetite control regions have been identified, including those in the arcuate nucleus (ARC), the periventricular nucleus (PVN) and the dorsomedial hypothalamic nucleus (DMH) (Valassi et al., 2008). These are sites of convergence and integration of many central and peripheral signals, not just macronutrients, that are involved in food intake and energy expenditure mechanisms, e.g., a group of neurons in the ARC stimulating food intake via neuropeptide Y (NPY) and agouti gene-related protein (AGRP). These neurons interact with those producing the anorexigenic pro-opiomelanocortin (POMC) and the cocaine/amphetamine-regulated transcript (CART) (Williams et al., 2001). Thus, a more comprehensive, unified model should include macronutrients as well as many single amino acids and other signaling molecules.

There are two distinguished types of food intake regulation: a) the short-term (satiety signals, SS) occurring at the beginning and end of a single meal; it also includes the length between meals and b) the long-term regulation (adiposity signal, AS) that is influenced by such factors as body fat deposition.

The SS providing information to the brain mainly send information to the nucleus of the solitary tract (NTS). These signals are generated in the GIT and abdominal viscera, as well as in the oral cavity and provide information about mechanical and chemical properties of food. The information is transmitted via vagal and spinal nerve to the NTS. The ASs arrive to the median eminence through ARC or through the blood-brain barrier (BBB). All these afferents are integrated in a complex and not fully understood network.

Hormones like leptin and insulin, both secreted into the blood, reflect the stored body fat. These hormones can pass the BBB and stimulate specific receptors. Hypothalamic areas are richly supplied by axons from ARC, which has greater concentrations of leptin and insulin receptors than any other hypothalamic site (Valassi et al., 2008).

The ARC exerts opposing actions on food intake responding not only to leptin and insulin, but also to gut hormones (the most studied are ghrelin and, recently, PYY). The neurophysiological pathways suggest that feeding is regulated by a feedback loop, where the hypothalamus provides the long-term regulatory input to the NTS, which acts as a setpoint (Williams et al., 2001).

It has recently been proposed that the ARC is required for the coordination of homeostatic circadian systems including temperature and activity. Authors tested this hypothesis by injecting saporin toxin conjugated to leptin into the ARC of rats. Wiater et al. showed that the leptin-sensitive network is required for entrainment of activity by photic cues and entrainment of temperature by food but is not required for entrainment of activity by food or temperature by photic cues (Wiater et al., 2013).

### Another player: the gastrointestinal tract and gut microbiota

The gastrointestinal tract (GIT) plays a central role in the control of energy balance. Many molecules produced by the GIT exert hunger or satiety effects on the brain. Ghrelin is a peptide produced mainly by the stomach's oxyntic cells that stimulates ghrelin secretion in the hypophysis and has some neuroendocrine activities. However, its orexigenic properties are the most relevant to us and ghrelin is the only known peripheral orexigenic hormone (Date, 2012). Cholecystokinin (CCK) is a peptide produced mainly in the duodenum and jejunum that acts on the vagus nerve and directly on the hypothalamic nuclei. CCK is an anorexigenic factor and it reduces food intake, meal size and duration (Murphy et al., 2006). Three other related hormones are pancreatic polypeptide (PP), amylin, and peptide YY (PYY). PP is a peptide produced by the endocrine pancreas in relation to the caloric content of meals, and it reduces food intake both in rodents and humans. Amylin is a peptide co-secreted with insulin; its main effect on food control is a reduction of meal sizes and food intake (Murphy et al., 2006). Peptide YY (PYY) is produced in the gut and is similar to PP. PYY is stored in intestinal cells and released into the circulation as PYY_3−36_, a truncated form of PYY. The release of PYY_3−36_ is dependent on a meal's caloric and fat content (Veldhorst et al., 2008). The glucagon-like peptide 1 (GLP-1) is produced by the cleavage of pro-glucagon gene in the intestine. It acts as incretin at a pancreatic level, promoting insulin secretion and as neuro hormone on hypothalamic nuclei, inducing satiety (Valassi et al., 2008).

The gut-brain link is important not only for the hormones produced by the gut, but also for the long-term body weight regulation. Studies in mice indicate that the gut microbiome influences both sides of the energy balance by contributing to nutrient absorption and regulating host genes that affect adiposity [however there are conflicting reports (Parks et al., 2013; Schele et al., 2013)]. However, it remains uncertain just how important gut microbiota are for nutrient absorption in humans. A cohort study has demonstrated that the nutrient load is a key variable that can influence the gut/fecal bacterial content over short time frames. Furthermore, the observed associations between gut microbes and nutrient absorption indicates a possible role of the human gut microbiota in the regulation of the nutrient intake and utilization (Jumpertz et al., 2011).

Moreover, according to recent evidence, meal onset appears to be biochemically induced only in the case of serious energy deprivation, while usually it is controlled by social, cultural and environmental factors strictly related to the lifestyle (Karatsoreos et al., 2013).

### Systemic ketosis in KD therapy

Ketogenic diets have become popular in recent decades for their demonstrated positive effects on weight loss (Bueno et al., 2013), though the precise mechanism of action is not fully understood (Paoli, 2014). In fact there is contradictory data about KD in mice and rats. In fact, there are contradictory data about KD in mice and rats. For example whilst a huge amount of data confirm that KD in humans is effective in weight reduction, improving lipidemia and glucose tolerance (Bueno et al., 2013), it has been recently demonstrated that a long-term KD (22 weeks) caused dyslipidemia, a pro-inflammatory state, hepatic steatosis, glucose intolerance and a reduction in beta and alpha cell mass, all without weight loss in mice (Ellenbroek et al., 2014). Two considerations should be made: (1) the induction of ketosis and the response to ketosis in humans and mice are quite different and (2) mice and humans have different life spans, and results obtained in mice after several weeks on the diet can correspond to months on the diet in humans (Demetrius, 2005, 2006).

Regardless of its efficacy for weight loss, the medium-long diet duration (Paoli et al., 2013) is over-cautiously received by the physicians, perhaps due to the lack of attention to the topic in specialized medical education courses. As a result, most physicians associate the term “ketosis” only in the context of diabetic ketoacidosis.

**Figure 1:**
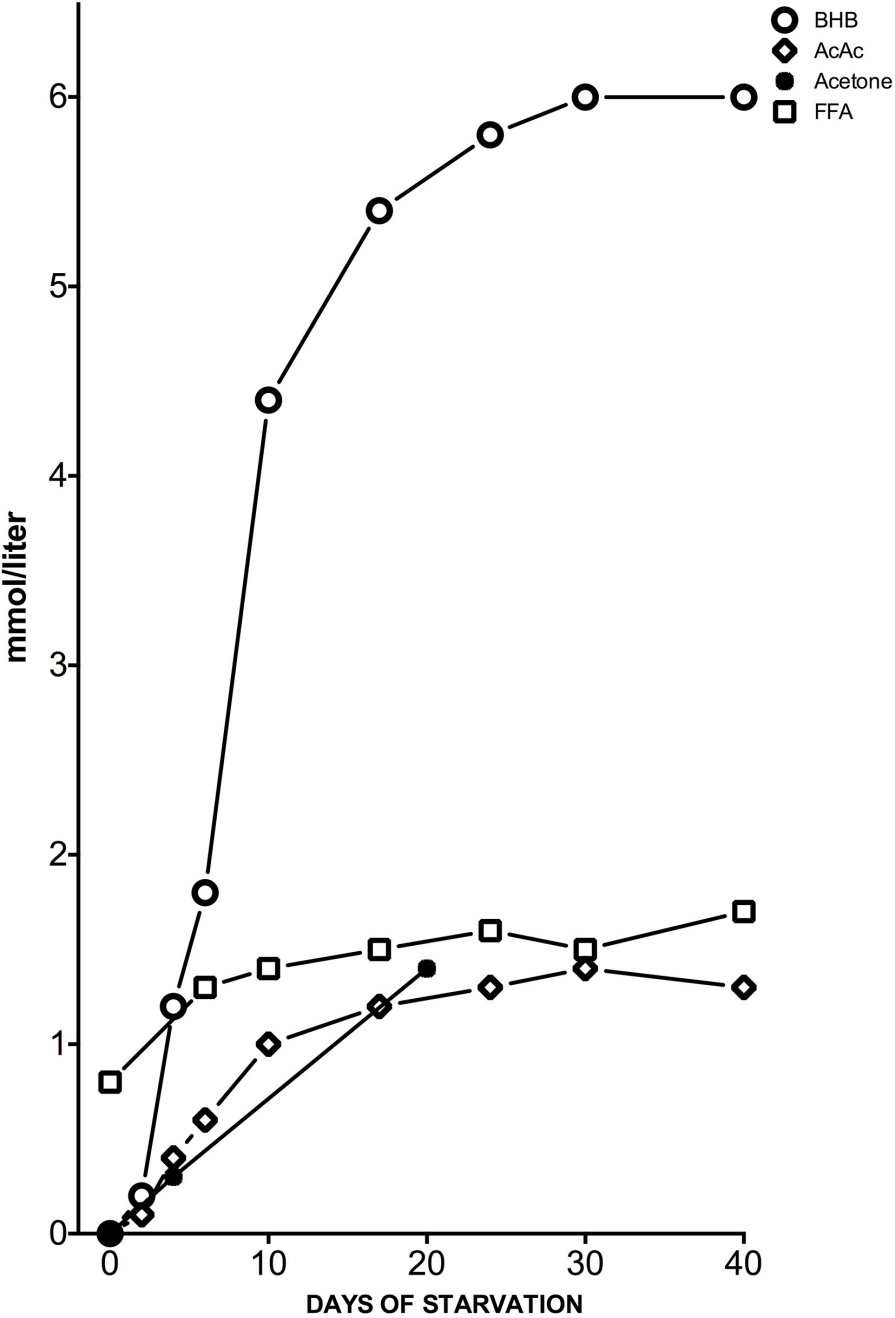
**Concentrations of KB: acetone, BHB and acetoacetic acid (AcAc), and plasma free FAs (FFA) from the post-absorptive state to 40 days of starvation in human subjects**. Y axis was expanded to better describe the great change in BHB concentration. Modified from Fukao et al. (2004), Owen (2005).

Meanwhile, the KD induces a ketosis that is not a pathological but physiological condition occurring on a daily basis. Hans Krebs was the first to use the term “physiological ketosis” despite the common view of it as oxymoron (Krebs, 1966); this physiological condition, i.e., ketosis, can be reached through fasting or through a drastically reduced carbohydrate diet (below 20 g per day). In these conditions, glucose reserves become insufficient both for normal fat oxidation via the supply of oxaloacetate in the Krebs cycle and for the supply of glucose to the central nervous system (CNS) (Felig et al., 1969; Owen et al., 1969) (Figure 1). It is well-known that the CNS cannot use FAs as an energy source because free FAs cannot cross the blood-brain barrier (BBB). This is why the brain normally uses only glucose. After 3–4 days without carbohydrate intake (KD or fasting) the CNS must find alternative energy sources as demonstrated by Cahill et al. (Owen et al., 1967, 1969; Felig et al., 1969; Cahill, 2006). These alternative energy sources are the ketones bodies (KBs): acetoacetate (AcAc), β-hydroxybutyric acid (BHB) and acetone and the process of their formation occurring principally in the mitochondrial matrix in the liver is called ketogenesis (Fukao et al., 2004). Usually the concentration of KB is very low (<0.3 mmol/L) compared to glucose (≅ 4 mmol) (Veech, 2004; Paoli et al., 2010). Since glucose and KB have a similar KM for glucose transport to the brain the KB begin to be utilized as an energy source by the CNS when they reach a concentration of about 4 mmol/L (Veech, 2004), which is close to the KM for the monocarboxylate transporter (Leino et al., 2001).

**Figure 2:**
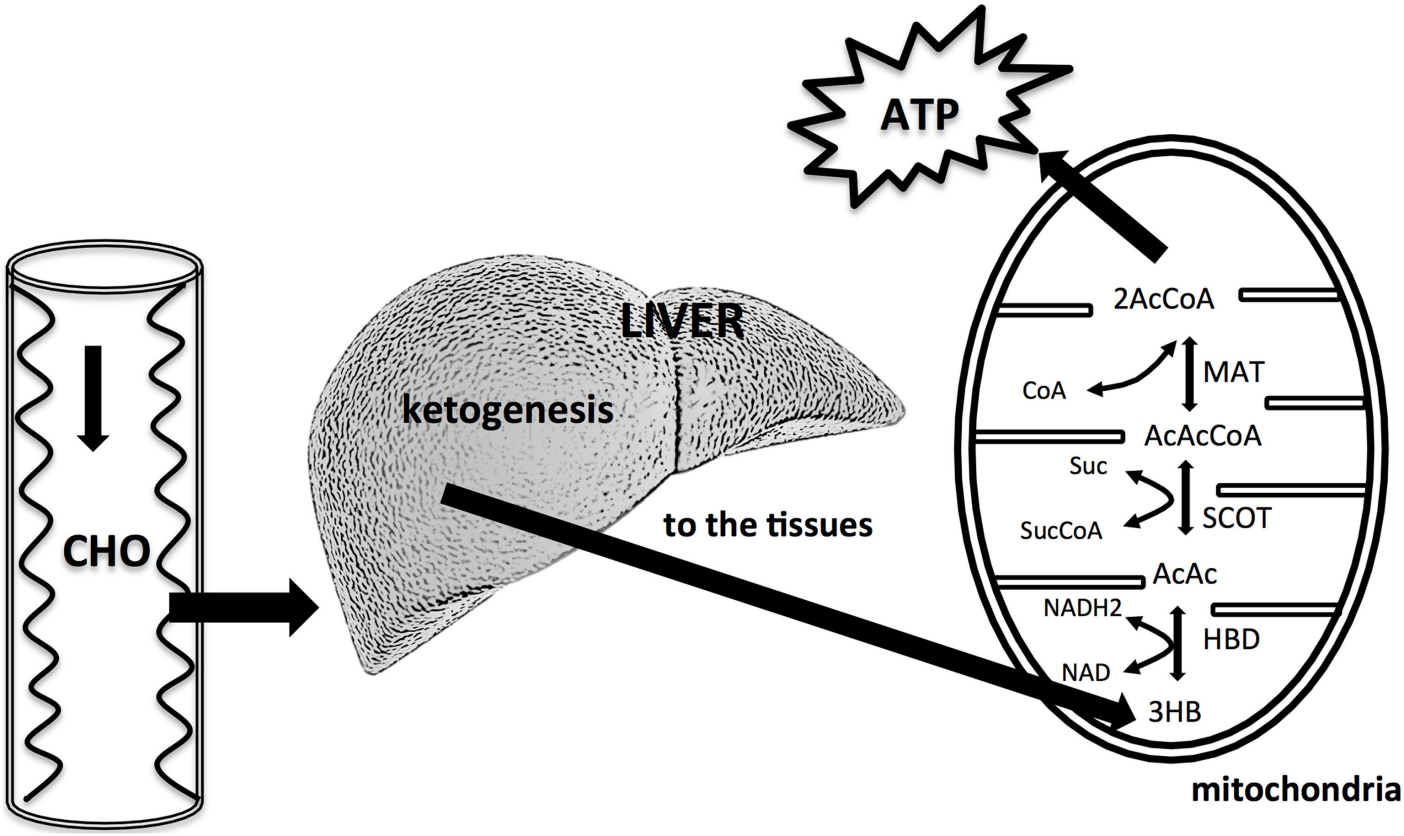
**A reduced availability of dietary carbohydrates leads to an increased liver production of KBs**. The liver cannot utilize KBs because it lacks the mitochondrial enzyme succinyl-CoA: 3-ketoacid (oxoacid) CoA transferase (SCOT) necessary for activation of acetoacetate to acetoacetyl CoA. KBs are utilized by tissues, in particularly by brain. KBs enter the citric acid cycle after being converted to acetyl CoA by hydroxybutyrate dehydrogenase (HBD), succinyl-CoA: 3–CoA transferase (SCOT), and methylacetoacetyl CoA thiolase (MAT). Modified from Owen (2005), Paoli et al. (2014).

KBs can cross the BBB but not in a homogenous manner. For example, past experiments have demonstrated that BHB utilization is different in various brain areas (Hawkins and Biebuyck, 1979). Areas without BBB, hypothalamic regions and the lower cortical layers have a higher BHB metabolism compared to the lower one of the basal ganglia (Hawkins and Biebuyck, 1979). Also the metabolic meaning of the three KBs is different: while the main KB produced in the liver is AcAc, the primary circulating ketone is BHB. The third one, acetone, is produced by spontaneous decarboxylation of AcAc, and it is the cause of the classic “fruity breath.” Acetone does not have any metabolic functions, but it can be used as a clinical diagnostic marker. BHB acid is not, strictly speaking, a KB because the ketone moiety has been reduced to a hydroxyl group. Under normal conditions the production of free AcAc is negligible and this compound, transported via the blood stream, is easily metabolized by various tissues including skeletal muscles and the heart. In conditions of overproduction, AcAc accumulates above normal levels and a part is converted to the other two KBs. The presence of KBs in the blood and their elimination via urine causes ketonemia and ketonuria. Apart from being the fundamental energy supply for CNS, glucose is necessary for the replenishment of the quota of oxaloacetate, since this intermediate of the tricarboxylic acid cycle (TCA) is labile at body temperature and cannot be accumulated in the mitochondrial matrix. Hence it is necessary to refurnish the TCA with oxaloacetate via the anaplerotic cycle that derives it from glucose through ATP dependent carboxylation of pyruvic acid by pyruvate carboxylase (Jitrapakdee et al., 2006). This pathway is the only way to create oxaloacetate in mammals. Once produced by the liver, KBs are used by tissues as a source of energy (Fukao et al., 2004; Veech, 2004; McCue, 2010): initially BHB is converted back to AcAc that is subsequently transformed into Acetoacetyl-CoA that undergoes a reaction producing two molecules of Acetyl-CoA to be used in the Krebs cycle (Figure 2).

It is interesting to note that the KB are capable of producing more energy than glucose due to the changes in mitochondrial ATP production induced by KB (Kashiwaya et al., 1994; Sato et al., 1995; Veech, 2004). During fasting or KD glycaemia, though reduced, remains within physiological levels (Seyfried and Mukherjee, 2005; Paoli et al., 2011). This euglycemic response to extreme conditions comes from two main sources: glucogenic amino acids and glycerol liberated via lysis from triglycerides (Vazquez and Kazi, 1994; Veldhorst et al., 2009). Glucogenic amino acids (neoglucogenesis from amino acids) are more important during the earlier phases of KD, while the glycerol becomes fundamental as the days go by. Thus, the glucose derived from glycerol (released from triglyceride hydrolysis) rises from 16% during a KD to 60% after a few days of complete fasting (Vazquez and Kazi, 1994). According to Bortz (1972) 38% of the new glucose formed from protein and glycerol is derived from glycerol in the lean while 79% in the obese (Bortz et al., 1972). It is important to note that during physiological ketosis (fast or very low calorie ketogenic diets) ketonemia reaches maximum levels of 7–8 mmol/L with no change in blood pH, while in uncontrolled diabetic ketoacidosis blood concentration of KBs can exceed 20 mmol/L with a consequent lowering of blood pH (Robinson and Williamson, 1980; Cahill, 2006) (Table 1).

**Table 1:** **Blood levels during a normal diet, ketogenic diet, and diabetic ketoacidosis (Paoli et al., 2012)**.

**Blood levels**	**Normal diet**	**Ketogenic diet**	**Diabetic ketoacidosis**
Glucose (mg/dL)	80–120	65–80	>300
Insulin (μU/L)	6–23	6.6–9.4	≅ 0
KB conc (mmol/L)	0.1	7–8	>25
pH	7.4	7.4	<7.3

We can say that no species, including humans, could have survived for millions of years without the ability to withstand brief periods of hunger or starvation (Amen-Ra, 2006). These periods of fasting are themselves ketogenic (McCue, 2010) during which the concentrations of insulin and glucose decrease while that of glucagon increases in the attempt to maintain normal blood glucose levels. When the body passes from a condition of food abundance to one of deprivation (or else via VLCKD simulated deprivation), there is, with a slight delay, an increase in the concentration of free FAs as well as KB in the blood. Thus, from this point of view KD could be compared to caloric restriction for fasting. These manipulations of nutrients, both in quantity and quality, seem to not only act on blood glucose/KB level but also to promote changes in metabolic pathways and cellular signaling. How this kind of metabolic condition (ketosis) can affect satiety and hunger mechanisms is still a matter of debate.

### Effects of ketosis on hunger and satiety

Although convincing, the bulk of evidence in relation to the inhibitory effects of ketosis on appetite is still anecdotal. Preliminary scientific reports seem to support this phenomenon, and the evidence shows that KD is more effective, at least in the short/medium-term, on fat loss (Paoli, 2014). It was demonstrated that diet-induced weight loss leads to changes in energy expenditure and in appetite-regulating hormones that facilitate weight regain and the return to initial energy homeostasis (Sumithran et al., 2011). This response to alteration of energy balance nullifies the success of many dietary approaches. It is well-known that the long-term success of a nutritional approach is defined by the amount of weight regain and is the main problem regarding the so-called weight cycling or “yo-yo” effect (Jeffery, 1996). A recent study by our group has demonstrated that a brief ketogenic period, if followed by a longer period of correct Mediterranean diet could avoid this yo-yo effect (Paoli et al., 2013). During the ketogenic period subjects reported less hunger, confirming previous studies (Nickols-Richardson et al., 2005; Johnston et al., 2006; Johnstone et al., 2008) on hunger-suppression effect of ketogenic diet. Despite these clinical findings, the mechanisms of action of ketosis on appetite reduction are still not completely understood. Clinical results are suggestive of both direct and indirect (via modifications of hunger-related hormones concentration) actions of KBs on appetite (Sumithran et al., 2013).

### Role of ketosis in nutrient-specific control of hungers and satiety

The findings of a stable (Chearskul et al., 2008) or slightly increased response (Sumithran et al., 2013) of post-prandial FFA after KD can be viewed in the nutrient-static context. Elevated circulating FFA may actually reduce food intake and glucose production through actions on specific hypothalamic neurons (Obici et al., 2003). It has been suggested that this effect could be mediated by the increase of cellular concentration of long-chain FAs-CoA in the arcuate nuclei of the hypothalamus (Obici et al., 2003).

Brain glucose and KB uptake was investigated in rats subjected to mild experimental ketonemia induced by 2 weeks on the KD or by 48 h fasting. To test this, researchers developed a carbon-11 labeled AcAc (11)C-AcAc for PET use. They found in rats that after 10 days of KD (11)C-AcAc brain uptake increased up to 8-fold, an increase comparable to those measured after 48 h of fasting (Pifferi et al., 2008).

The BBB, largely formed by the brain capillary endothelial cells, provides a protective barrier between the systemic blood and the extracellular environment of the CNS. Passage of FAs from the blood to the brain may occur either by diffusion or by proteins that facilitate their transport. Studies indicate that FATP-1 and FATP-4 are the predominant FA transport proteins expressed in the BBB based on human and mouse expression studies (Mitchell et al., 2011).

As a matter of fact, in animal models intracerebroventricular injections of long-chain FA reduced hypothalamic expression of NPY. NPY is an important orexogenic neuropeptide that is a downstream target of leptin and insulin in the hypothalamus. In some forms of hyperphagic obesity, characterized by elevated plasma leptin and insulin levels, the lack of action of insulin on NPY expression could explain the pathological condition. Central administration of oleic acid, fatty-acid synthase, or CPT-1 inhibitors prevents the rise in hypothalamic NPY mRNA induced by fasting (Obici et al., 2003). But glucose level is also involved in KD's food control mechanisms. According to glucostatic theory (Mayer, 1955) data indicates that ketosis did not influence FA glucose but instead stimulated the elevation of post-prandial glucose (Sumithran and Proietto, 2013) in non-diabetic subjects, while in diabetics there was a reduction of fasting glucose (Westman et al., 2008). It is important to note that carbohydrate availability may increase cellular levels of long-chain FA-CoA through an increase of malonyl-CoA, which inhibits oxidation of FAs.

Another product of elevated levels of free FA is polyunsaturated FA (PUFA). The potential ability of PUFA to block seizure activity in the brain is speculated to be associated with KD. Some mechanisms are thought to be a direct inhibition of voltage-gated sodium and calcium channels, modulation of a lipid-sensitive potassium channel, the activity of the sodium pump to limit neuronal excitability, or the induction of expression and activity of proteins in the mitochondria, thereby inducing a neuroprotective effect by partially inhibiting the production of reactive oxygen species (ROS) (Bough and Rho, 2007; Paoli et al., 2014).

### Role of ketosis on neuroendocrine control of hungers and satiety

The discovery of many appetite-related hormones provided molecular basis for appetite control, decreasing the relevance of the metabolites hypothesis (Karatsoreos et al., 2013). Recently, Sumithran et al. demonstrated that there is a long-term persistence of changes in some peripheral hormones involved in food control (Sumithran et al., 2011). In this study, they found a significant difference in mean levels of many food intake-related hormones 1 year after the cessation of weight loss via the hypocaloric diet. There was a long lasting decrease of anorexigenic compounds: leptin, PYY, cholecystokinin, insulin, and pancreatic peptide and an increase of the orexigenic molecule ghrelin. Moreover, they found that hunger remained elevated 1 year after diet cessation. In a successive study the same group investigated hunger-related hormones after 8 weeks of KD, demonstrating that during ketosis the increase of ghrelin (a strong stimulator of appetite) was suppressed (Sumithran et al., 2013). These results are consistent with those of Ratliff et al (Ratliff et al., 2009), who found no significant change in fasting plasma ghrelin after 12 weeks of VLCD.

Moreover, in the above study of Sumithran et al. (2013), ketosis maintains post-prandial secretion of CCK as previously demonstrated by other researchers (Chearskul et al., 2008). Note that the orexigenic effect of BHB is blocked by transection of the common hepatic branch of the vagus nerve (Langhans et al., 1985). The hepatic branch contains fibers from the proximal small intestine, stomach and pancreas, and is sensitive to CCK (Horn and Friedman, 2004); ghrelin signals to brain are also transmitted via vagus nerve (Habara et al., 2014). Thus, the effects of ketosis on these two appetite-related hormones could be one of the many factors related to the effects of such nutritional regimen on food control.

Many questions about the role of such an important intermediate of lipid metabolism remains unanswered, e.g., the role of BHB in food control. For example, whether or not BHB could act as a satiety signal in the brain, considering its role in energy supply to CNS. We have to consider that the effects of KBs on hunger reduction can only be seen after many days following fasting or KD initiation (Paoli et al., 2010); this is consistent with the abovementioned threshold of brain utilization of KB as an energy source, i.e., 4 mmol/L (Veech, 2004), which is close to the Km for the monocarboxylate transporter (Leino et al., 2001). During the first days of fasting or KD there is a rise of BHB and adiponectin concentrations (Halberg et al., 2005). One of the putative causes of hunger in starved humans may be due—together with other causes—to adiponectin. When adiponectin binds to its receptor AdipoR1, AMP-activated protein kinase (AMPK) is phosphorylated in the ARC of the hypothalamus (Valassi et al., 2008). The increase of AMPK activity in the hypothalamus may increase food intake and hepatic glucose output in mice while the decrease seems to reduce food intake (Zhang et al., 2009). KDs can also act similarly to a caloric restriction on AMPK (Newman and Verdin, 2014). Interestingly, AMPK seems to have opposing actions on the liver, muscle tissues and the brain: in liver and muscle AMPK activation increases FA oxidation by decreasing malonyl-CoA concentrations (Malonyl-CoA is the first intermediate in the lipogenic pathway and is also an inhibitor of carnitine palmitoyltransferase-1 (CPT-1). CPT-1 activity can be limiting for FA oxidation), through the inactivation of the acetyl-CoA carboxylase 1 (ACC1). AMPK can also increase the activity of malonyl-CoA decarboxylase (MCD), which enhances the decrease of malonyl-CoA levels.

**Figure 3:**
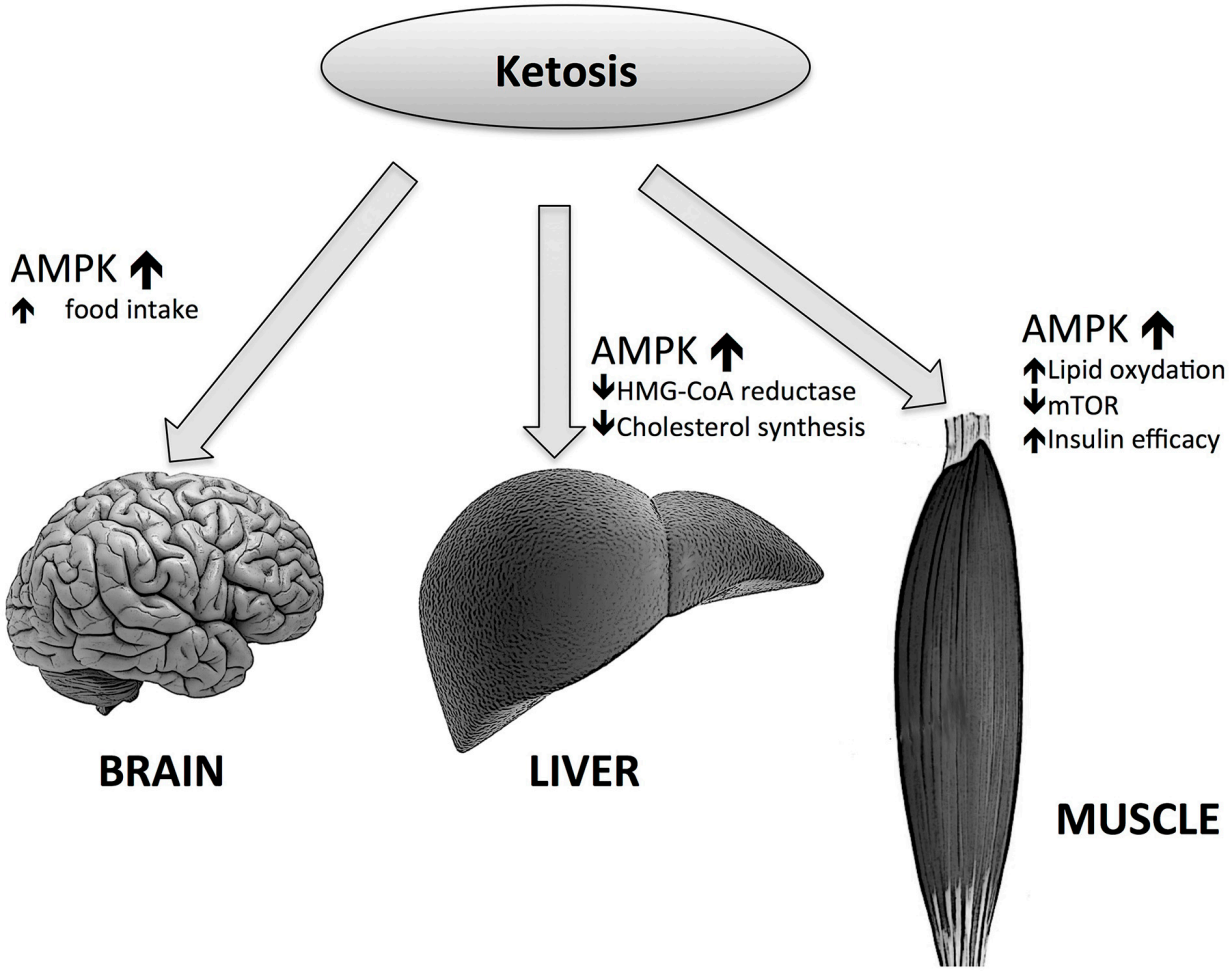
**Effects of ketone bodies on AMP-activated protein kinase (AMPK) actions in different tissues**.

On the contrary, in the brain, as mentioned above, the increase of AMPK activity leads to higher food intakes. But the effect of AMPK in the brain is more complicated; mice lacking AMPKa2 in pro-opiomelanocortin neurons develop obesity, while the deficiency of AMPKa2 in agouti-related protein neurons results in an age-dependent phenotype. Thus, the conclusion is that even while AMPK is a regulator of hypothalamic functions, it does not act as a signal for energy deficit or excess (Claret et al., 2007). However, the picture is more complex than this (Figure 3); BHB induces AgRP expression while increasing ATP and inhibiting AMPK phosphorylation (Cheng et al., 2008). Moreover, Laeger and colleagues have recently demonstrated that under physiological conditions BHB decreases AMPK phosphorylation and AgRP mRNA expression in GT1-7 hypothalamic cells (Laeger et al., 2012).

Another mechanism that could be involved in food-regulation during KD is the gamma aminobutyric acid (GABA) and glutamate regulation. Wu et al. demonstrated that GABAergic signaling from the NPY/AgRP neurons to the parabrachial nucleus (located in the dorsolateral part of the pons) is involved in many regulatory sensory stimuli including taste and gastric distension, regulate feeding behavior. GABA signaling seems to prevent animals from anorexia when AgRP neurons were destroyed (Wu et al., 2009). These findings are yet another contradictory aspect of KDs and food behavior; ketosis should increase the availability of glutamate (via diminution of transamination of glutamate to aspartate) and therefore increase GABA and glutamine levels; moreover, in ketosis, the brain imports a huge amount of acetate and converts it through glia into glutamine (an important precursor of GABA) (Yudkoff et al., 2008). The result of these mechanisms, together with the increased mitochondrial metabolism and flux through the TCA cycle, is an increased synthesis of glutamine and a “buffering” of glutamate. These results are not consistent with the well-documented anorexigenic effect of KDs, and therefore the GABA hypothesis cannot be taken into account despite the mild euphoria often reported during a KD that is probably due to the action of BHB (Brown, 2007) and can help to reduce appetite.

## Other possible mechanisms

### Gut microbiota

It is known that different dietary components exert some effects on gut microbiome composition, mainly in relation to obesity and inflammatory states. In general, a Mediterranean diet has a positive effect while a high-protein diet seems to have detrimental effects due to putrefaction phenomena (Lopez-Legarrea et al., 2014; Flint et al., 2015). Few data are available at this time about the effects of KD on gut microbiota. For example, a study by Crawford et al. (2009) investigated the regulation of myocardial ketone body metabolism by the gut microbiota and demonstrated that, during fasting, the presence of gut microbiota improved the supply of ketone bodies to the heart where KBs were oxidized. In the absence of a microbiota, low levels of KB was associated with a related increase in glucose utilization, but heart weight was still significantly reduced. The myocardial-mass reduction was completely reversed in germ-free mice feeded with a ketogenic diet. Regarding food control we can hypothesize that the particular metabolic state of ketosis could provide some benefit to weight and food control via synergic actions between butyrate production by gut bacteria and circulating high blood ketones (Sanz et al., 2015).

### Reactive oxygen species

As is in the case of GABA, the intracellular reactive oxygen species (ROS) hypothesis works against the hunger-suppressive role of KD: it has been demonstrated that the hypothalamic ROS increase through NADPH oxidase is required for the eating-inhibitory effect of insulin (Jaillard et al., 2009); moreover it has been demonstrated that there is a ROS-dependent signaling pathway within the hypothalamus that regulates the energy homeostasis, and that activation of ROS-sensitive mechanisms could be sufficient to promote satiety (Benani et al., 2007). On the other side, KBs decreases mitochondrial production of ROS by increasing NADH oxidation in the mitochondrial respiratory chain (Maalouf et al., 2007).

## Conclusions

**Figure 4:**
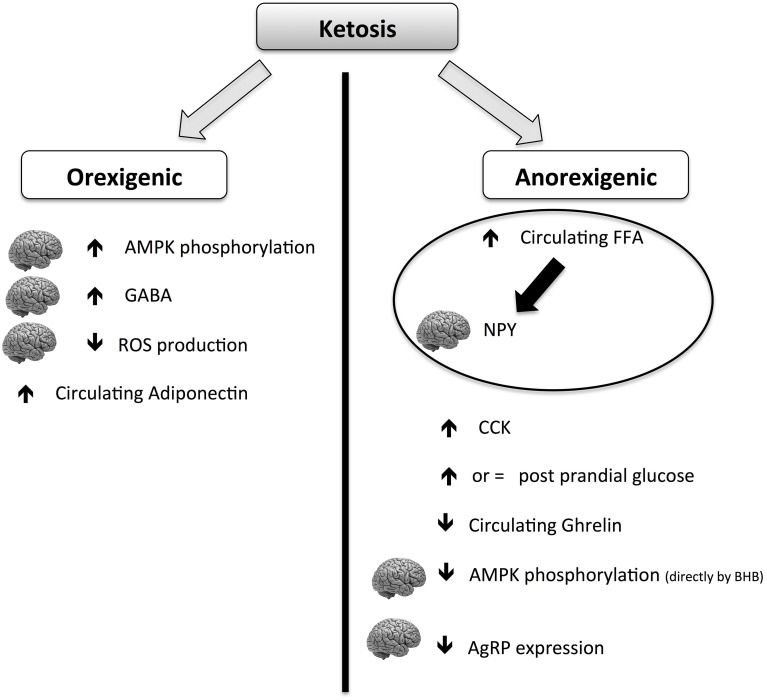
**Scheme of orexigenic and anorexigenic effects of ketosis**. The picture is highly schematic. For more details please see the text. AMPK, AMP-activated protein kinase; CCK, cholecystokinin; GABA, gamma-aminobutyric acid; BHB, β-hydroxybutyric acid; FFA, free fatty acids; ROS, reactive oxygen species; NPY, neuropeptide Y; AgRP, agouti gene-related protein.

Although the hunger-reducing effect of KD is well-documented, its main mechanisms of action are still elusive. The global picture is complicated by the contradictory role of ketosis on anorexigenic and orexigenic signals (summarized in Figure 4). Ketones (mainly BHB) can act both orexigenically or anorexigenically. In the orexigenic mechanism, it increases the circulating level of adiponectin, increasing brain GABA and AMPK phosphorylation and decreasing brain ROS production. The anorexigenic mechanism triggers a main normal glucose meal response, increasing circulating post-meal FFA (thus reducing cerebral NPY), maintaining CCK meal response and decreasing circulating ghrelin. It can be postulated that the net balance of the contrasting stimuli results in a general reduction of perceived hunger and food intake. More studies are needed to explore the mechanism of potential beneficial effects of KD on food control.

### Conflict of interest statement

The authors declare that the research was conducted in the absence of any commercial or financial relationships that could be construed as a potential conflict of interest.
